# Role of fundoplication in treatment of patients with symptoms of hiatal hernia

**DOI:** 10.1038/s41598-019-48740-x

**Published:** 2019-08-29

**Authors:** Zhi-tong Li, Feng Ji, Xin-wei Han, Li-li Yuan, Zheng-yang Wu, Miao Xu, De-lu Peng, Zhong-gao Wang

**Affiliations:** Department of Interventional Radiology, The First Affiliated Hospital, Zhengzhou University, No. 1, East Jian She Road, Zhengzhou, 450052 Henan Province People’s Republic of China

**Keywords:** Gastro-oesophageal reflux disease, Oesophagogastroscopy

## Abstract

Gastroesophageal reflux disease (GERD) is often associated with hiatal hernia (HH). However, the need for fundoplication during hiatal hernia repair (HHR) remains controversial. The objective of this study was to evaluate the effect of HHR with concomitant laparoscopic Nissen fundoplication (HHR-LNF) in HH patients. A total of 122 patients with symptomatic HH were randomized to receive either HHR (n = 61) or HHR-LNF (n = 61). The measures of evaluating outcomes included DeMeester scores (DMS), complications, Reflux Diagnostic Questionnaire and patients’ satisfaction 24 months following surgery. Despite comparable values in both groups at randomization, the DMS, total numbers of reflux episodes and percentage of time with pH < 4 were significantly higher in HHR group than in HHR-LNF group (*P* = 0.017, *P* = 0.002 and *P* = 0.019, respectively) at 6 months after surgery. One months postoperatively, complications were higher in the HHR-LNF group than in the HHR group (all *P* < 0.001), and there was no difference between the two groups at 6 months. By the end of the 2-year follow-up, HHR-LNF group showed a significantly lower reflux syndrome frequency-intensity score and greater percentage of satisfaction compared with HHR group (all *P* < 0.001). Laparoscopic HHR should be combined with a fundoplication in GERD patients with HH. HHR-LNF is safe and effective, not only improve reflux-related symptom, but also reduce the incidence of complications.

## Introduction

The history of antireflux surgery over the last 48 years (Allison^1^) has shown that reduction of the hiatal hernia in conjunction with diaphragmatic crural approximation as shown by Allison is at best a transient antireflux deterrent and that an additional procedure needs to be performed. Over time, that procedure has been a posterior gastroprexy as described by Hill^2^ with little or no fundoplication. Ultimately, a fundoplication is performed either through the chest (Belsey Mark IV) or abdomen involving varying degrees of encirclement of the distal esophagus by the fundoplication. Laparoscopic hiatal hernia repair (HHR) has been shown to provide good short- and long-term results in gastroesophageal reflux disease (GERD)^3,4^, and may reduce the laparoscopic antireflux surgery complications^5^. Fundoplication is also an important component of laparoscopic antireflux surgery performed for medication-refractory GERD^6^.

However, whether fundoplication should be a routine adjunct to HHR in patients with hiatal hernia (HH) is still controversial. A blinded randomized controlled study showed laparoscopic repair of paraesophageal hiatal hernias should be combined with a fundoplication to avoid postoperative GERD and concomitant esophagitis^7^. Postoperative GERD was observed in 20% and 34% of patients with paraesophageal hiatal hernias repair without fundoplication^8,9^, and 32% new onset of GERD at a mean follow-up of 3 years^10^. Some people who are not recommended fundoplication think there are risk of fundoplication-related complications and side effects. 58% of patients have gas bloat syndrome following fundoplication^11^, and about 20% of patients have reported new-onset complaints after fundoplication^12,13^, even as many as 96%^14^.

Despite the above history of surgical advancement, we feel a re-examination of diaphragmatic crural approximation alone and combined with the best procedure to date, a 360° fundoplication as performed would be meaningful and necessary.

In this study, laparoscopic HHR (the effect of hernia reduction and only repair of diaphragmatic crura) was compared to HHR with concomitant fundoplication in terms of relative frequency and severity of symptoms before laparoscopic antireflux surgery, and DeMeester scores (DMS), relative symptom improvement, patients’ satisfaction and complications after antireflux surgery.

## Materials and Methods

### Patient data

Between December 2013 and December 2015, 136 consecutive patients with symptomatic HH were considered for inclusion in this study. Patients were eligible for enrollment in this study if they had persistent symptoms despite daily use of proton-pump inhibitors (omeprazole 20 mg twice a day) for at least 3 months before surgery^15^. Patients were excluded if they had history of previous esophagogastric surgery, achalasia, Zollinger-Ellison syndrome, or malignant tumor. Patients failing to answer more than 40% of the questionnaire and those having a serious intercurrent event (e.g., autoimmune disease, coagulation disorder) during the course of the study were also excluded from the final analysis.

All patients underwent preoperative diagnostic evaluation with endoscopy, barium sulfate swallow radiology, and esophageal manometry. GERD was diagnosed if the patient had typical heartburn and regurgitation, endoscopic evidence of esophagitis or abnormal esophageal pH, reflux events and DMS (composite score) ≥ 14.72^16,17^. The following parameters were also evaluated: (1) the total number of reflux episodes, (2) total time for pH < 4, (3) time percentage of upright position pH < 4, (4) time percentage of supine position pH < 4, and (5) number of times pH < 4 for lasting 5 minutes. The HH were classified according to internationally accepted criteria^5^.

HH was classified into four types according to international standards, including type I (sliding), type II (pure paraesophageal), type III (mixed), and type IV (mixed with others organs rather than only gastric hernia sac content)^5^. Esophagitis was graded according to the Los Angeles (LA) classification^18^.

### Surgical technique

The surgical technique of laparoscopic surgical procedure was performed as described in our previous study^15^. The patient was sedated and placed in a reverse-trendelenburg position, with the lower extremities abducted. The operating surgeon stood between the legs of the patient, with the first assistant on the left side of the patient and the second assistant on the right side of the surgeon. A veress needle was inserted close to the rib cage, and a pneumoperitoneum was created. The primary 10-mm trocar was inserted at the upper edge of the navel. Four more trocars were then inserted in the upper abdomen under direct visualization. The liver was retracted, and the esophageal hiatus was exposed. The stomach was repositioned, and the hernia sac was dissected and reduced from the mediastinum using a harmonic scalpel. A 3–4 cm of esophageal reposition was dissected intra-abdominally. The diaphragmatic crurae were exposed, and sutured 2–3 times intermittently with non-absorbable lines to reduce the esophageal hiatus. When the hernial sac was >5 cm in size, a horseshoe-shaped polypropylene mesh patch was fixed around the esophagus from behind with 8–12 staples toward the diaphragm. If 360-degree Nissen fundoplication was added. A full-circle valve was formed from the posterior and anterior aspects of the fundus and sutured together using three separate nonabsorbable 2–0 sutures.

### Evaluation of GERD

During postoperative follow-up, objective evaluation included 24-hour esophageal pH monitoring and esophageal manometry at 6 months, as well as gastroscopy at 12 months. The 24-hour esophageal pH monitoring was assessed by DMS and percentage of time with pH < 4. Esophageal manometry was performed for measuring hypotonia of the lower esophageal sphincter and HH. The lower esophageal sphincter resting pressure (LESP), using mid-respiratory gastric pressure as zero reference, was measured mid-respiratory resting pressure in the high-pressure zone. Gastroscopy was used to identify recurrent HH and esophagitis. The criterion for gastroscopic diagnosis of recurrent HH was cephalic displacement of the proximal margin of the gastric mucosal folds by more than 2 cm in relation to the hiatus.

Questionnaires were completed at admission to investigate the baseline symptom scores, and mailed questionnaire were used during follow-up. Heartburn and regurgitation were the most typical symptom of GERD, and the occurrence of these symptoms were considered to indicate recurrence. A 6-point scale was applied to assess the severity and frequency of heartburn and regurgitation according to the Reflux Diagnostic Questionnaire (RDQ)^15,19^. Frequency of symptoms was graded as 0 (none), 1 (less than once per week), 2 (once or twice per week), 3 (three or four times per week), 4 (five or six times per week), and 5 (more than six times per week). Severity was graded as 0 (none), 1 (slight), 2 (mild), 3 (moderate), 4 (severe), and 5 (extremely severe). The sum of the frequency score and the severity score was designated as the symptom score.

Patients, satisfaction with the result of surgery was also assessed. Four grades were possible: excellent (complete resolution of symptoms); good (symptoms occurring once per month or less frequently); fair (symptoms occurring weekly or less frequently); and poor (symptoms occurring daily or more often, or as severe as that prior to surgery)^20^.

### Statistical analysis

Data analysis was performed using SPSS version 13.0 software (SPSS Inc, Chicago, IL, USA) and GraphPad InStat, version 3.06 (GraphPad Software, San Diego, CA, USA). Continuous variables were expressed as median or means ± standard deviations. Comparisons were made between HHR patients and HHR-LNF patients and between preoperative and postoperative status using the chi-squared test, the Wilcoxon paired-samples test, or the *t* test as appropriate. All statistical tests were two-sided. *P* < 0.05 was considered statistically significant.

### Ethics

The Ethics Committee of Zhengzhou University approved the study. The committee that approved the research, confirm that all research was performed in accordance with relevant guidelines/regulations. Written informed consent was obtained from all participants and/or their legal guardians.

## Results

**Figure 1:**
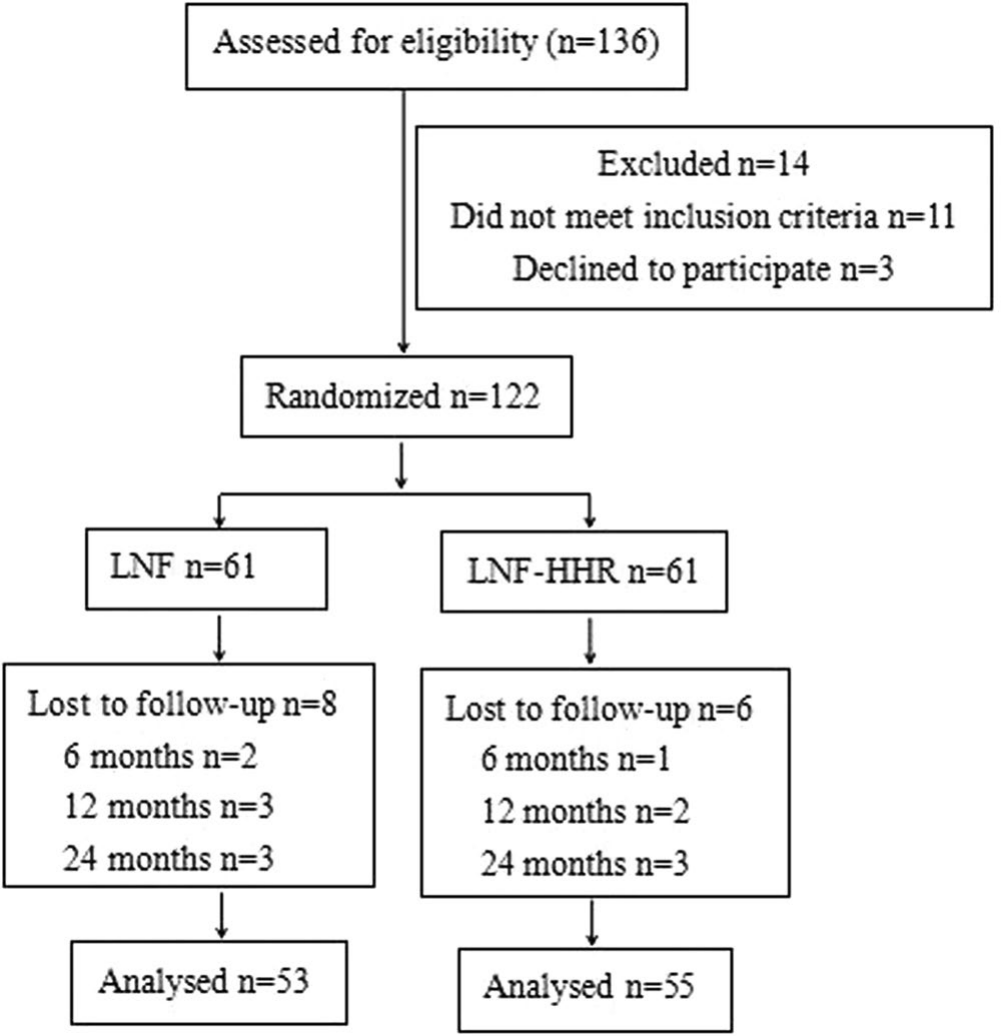
HHR vs. HHR-LNF flow diagram. HHR = laparoscopic hiatal hernia repair; HHR-LNF = laparoscopic hiatal hernia repair with concomitant Nissen fundoplication.

**Figure 2:**
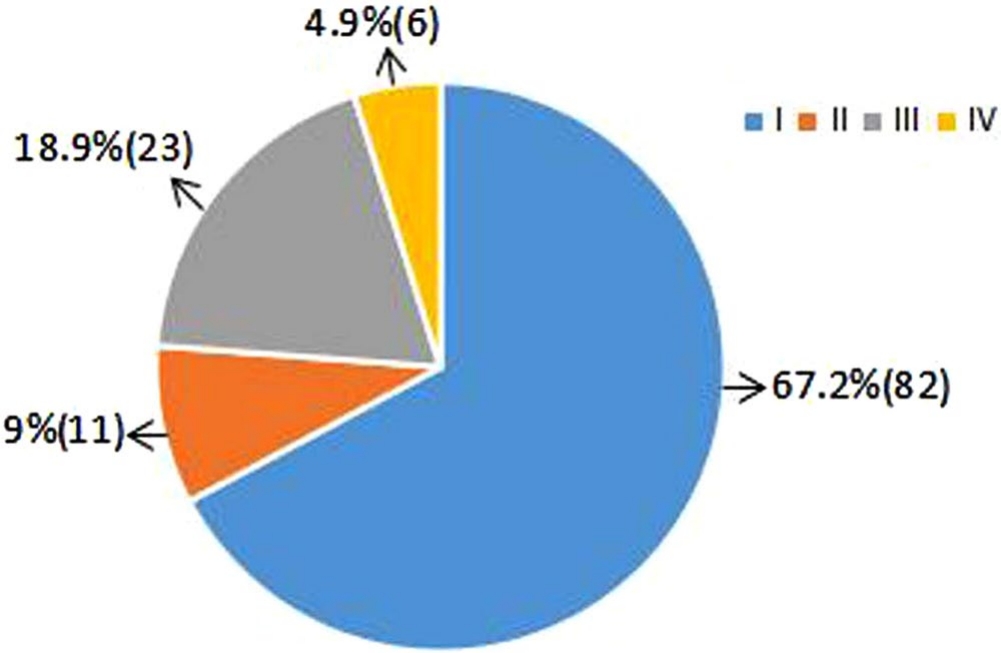
The types of hiatal hernia.

Of the total 136 patients, 14 patients did not meet inclusion criteria. The remaining 122 patients were randomly assigned into a HHR group (n = 61) or a HHR-LNF group (n = 61). The study flow was shown in Figs 1 and 2. Figure 1 showed the number of patients included in the follow-up. Figure 2 showed type I hernia was the most common type of hiatal hernia. The demographic and clinical characteristics of the patients were comparable between the two groups at baseline (Table 1). After 24-month follow-up, 53 of 61 (86.9%) LNF patients and 55 of 61 (90.2%) HHR-LNF patients were taken into account in the final analysis (Fig. 1).

**Table 1:** Comparison of demographic data between the HHR and HHR-LNF groups.

Characteristics	HHR (n = 61)	HHR-LNF (n = 61)	*P* value
Age, y, mean ± SD	52.3 ± 11.8	53.0 ± 11.7	0.735
Male, n (%)	31 (50.82)	36 (59.02)	0.270
BMI, kg/m^2^, mean ± SD	23.7 ± 3.9	23.8 ± 4.2	0.866
Esophagitis (n)	20	22	0.703
LA, A	10	11	0.810
LA, B	6	6	1
LA, C	3	4	0.697
LA, D	1	1	1
Type of hernia	10		
I, n	40	42	0.700
II, n	6	5	0.752
III, n	13	10	0.487
IV, n	2	4	0.402
DMS, mean ± SD	54.1 ± 24.3	52.4 ± 22.5	0.690
N. of reflux episodes	94.9 ± 39.9	106.0 ± 36.3	0.111
total time pH < 4, min	261.0 ± 92.9	268.1 ± 79.5	0.650
% time pH < 4	18.0 ± 6.1	18.6 ± 5.5	0.551
% time upright pH < 4	7.1 ± 3.0	7.3 ± 3.5	0.741
% time supine pH < 4	12.1 ± 7.0	11.3 ± 3.9	0.476
N. lasting 5 minutes reflux	10.5 ± 5.7	10.7 ± 6.3	0.869
LESP, mmHg, mean ± SD	4.6 ± 6.8	4.3 ± 6.5	0.788
UESP, mmHg, mean ± SD	66.5 ± 16.0	64.8 ± 15.7	0.548
Heartburn, mean ± SD	5.5 ± 1.8	5.6 ± 1.7	0.878
Regurgitation, mean ± SD	5.1 ± 1.7	5.5 ± 1.6	0.276

HHR, laparoscopic hiatal hernia repair; HHR-LNF, laparoscopic hiatal hernia repair with concomitant Nissen fundoplicationr; SD, standard deviation; LA, Los Angeles classification; DMS, DeMeester score; N, number; UESP, upper esophageal sphincter pressure (normal range: 34–104 mmHg); LESP, lower esophageal sphincter pressure (normal range: 13–43 mmHg).

### Intra- and postoperative complications

All procedures were completed successfully by laparoscopic methods. The mean operating time was shorter in the HHR group than in the HHR-LNF group (*P* < 0.001). Table 2 showed there was no significant difference between the groups in the mean duration of postoperative hospital stay (*P* = 0.305). One months postoperatively, patients in the HHR-LNF group experienced complications such as dysphagia, abdominal bloating, abdominal pain were higher than in the HHR group (*P* < 0.001; Table 2). Six months postoperatively, Table 2 showed there was no difference between the two groups in the incidence of the complications.

**Table 2:** Comparison of data between the HHR and HHR-LNF groups.

Characteristic	HHR (n = 61)	HHR-LNF (n = 61)	*P* value
Operating time (min)	85.6 ± 6.4	90.9 ± 12.3	0.003
Postoperative hospital stay (days)	3.5 ± 1.8	3.3 ± 1.4	0.305
Death, n	0	0	—
**postoperative complications**			
dysphagia			
1 month, n	12	25	0.004
6 months, n*	1	2	0.579
abdominal bloating			
1 month, n (%)	14	30	0.003
6 months, n*	2	5	0.252
abdominal pain			
1 month, n	6	15	0.031
6 months, n*	1	3	0.326

HHR, laparoscopic hiatal hernia repair; HHR-LNF, laparoscopic hiatal hernia repair with concomitant Nissen fundoplication; *three patients (two in the HHR group and one in the HHR-LNF group) were lost to follow-up at 6 months.

### Objective outcomes

Six months postoperatively, 54.24% (32/59) HHR patients and 63.33% (38/60) HHR-LNF patients were available for esophageal manometry and 24-hour pH monitoring. In some patients, esophageal manometry and 24-hour pH monitoring could not be performed again because of intolerance of the procedure and inspection fees. Significant improvement in DMS, the total numbers of reflux episodes, the total time for pH < 4, percentage of time with pH < 4, percentage time for pH < 4 in the upright position, percentage time for pH < 4 in the supine position, number of times for pH < 4 lasting 5 minutes and LESP were seen in both groups (*P* < 0.05 in all cases; Tables 1 and 3). The mean DMS, total numbers of reflux episodes, and the percentage of time with pH < 4 decreased from 54.1 ± 24.3, 94.9 ± 39.9, and 18.0% ± 6.1% to 12.7 ± 10.1, 45.8 ± 24.4, and 6.9% ± 5.9%, respectively, after HHR; and from 52.4 ± 22.5, 106.0 ± 36.3, and 18.6% ± 5.5% to 7.7 ± 6.8, 28.7 ± 20.7, and 4.0% ± 4.4%, respectively, after HHR-LNF (all *P* < 0.001 in both groups). However, the DMS, total numbers of reflux episodes and percentage of time with pH < 4 were significantly higher in HHR group than in HHR-LNF group (*P* = 0.017, *P* = 0.002 and *P* = 0.019, respectively; Table 3). LESP increased in both groups: from 4.6 ± 6.8 mmHg to 12.5 ± 8.0 mmHg after HHR, and from 4.3 ± 6.5 to 16.7 ± 7.8 mmHg after HHR-LNF (*P* < 0.001 in both groups; Tables 1 and 3). However, LESP was significantly higher in HHR-LNF group than in HHR group (*P* = 0.031; Table 3).

**Table 3:** Comparison of outcomes in the HHR and HHR-LNF groups at 6 months after surgery.

Variable	HHR (n = 32)	HHR-LNF (n = 38)	*P* value
DMS, mean ± SD	12.7 ± 10.1	7.7 ± 6.8	0.017
N. of reflux episodes	45.8 ± 24.4	28.7 ± 20.7	0.002
total time pH < 4, min	101.2 ± 84.0	49.8 ± 48.9	0.002
% time pH < 4	6.9 ± 5.9	4.0 ± 4.4	0.019
% time upright pH < 4	2.4 ± 2.0	1.1 ± 1.3	0.003
% time supine pH < 4	4.5 ± 4.3	2.9 ± 2.5	0.008
N. lasting 5 minutes reflux	4.1 ± 6.0	0.8 ± 1.8	0.002
LESP, mmHg, mean ± SD	12.5 ± 8.0	16.7 ± 7.8	0.031

HHR, laparoscopic hiatal hernia repair; HHR-LNF, laparoscopic hiatal hernia repair with concomitant Nissen fundoplication; DMS, DeMeester score; N, number; SD, standard deviation; LESP, lower esophageal sphincter pressure (Normal range: 13–43 mmHg).

At 12 months after surgery, 114 patients were available for gastroscopy. Table 4 showed esophagitis was documented in 44.64% (25/56) HHR group and 13.79% (8/58) HHR-LNF group (*P* < 0.001). Compared with the preoperative findings, the frequency of esophagitis was significantly lower after HHR-LNF (*P* = 0.005). The frequency of esophagitis was high after HHR without LNF, although the change was not statistically significant (*P* = 0.118; Table 4).

**Table 4:** Comparison of gastroscopy results.

Variable	Preoperatively		12 months after surgery		*P value	**P value	
	HHR (n = 61)	HHR-LNF (n = 61)	HHR (n = 56)	HHR-LNF (n = 58)	postoperatively 12 month	HHR	HHR-LNF
Esophagitis, n	20	22	25	8	0.00	0.188	0.005
LA, A	10	11	15	6	0.024	0.171	0.231
LA, B	6	6	8	2	0.041	0.459	0.164
LA, C	3	4	2	0	0.146	0.719	0.047
LA, D	1	1	0	0	—	0.336	0.327
HH, n							
I, n	40	42	1	0			
II, n	6	5	0	0			
III, n	13	10	2	1			
IV, n	2	4	0	0			

HHR, laparoscopic hiatal hernia repair; HHR-LNF, laparoscopic hiatal hernia repair with concomitant Nissen fundoplication; *P value is for comparison between HHR and HHR-LNF; **P values are for comparisons beteen preoperative and postoperative results; LA, Los Angeles Classification.

In Table 4, a recurrent HH was revealed in 1.7% (1/58) HHR-LNF group (1 type III hernias) and in 5.4% (3/56) HHR group (1 type I hernia and 2 type III hernia).

### Symptom outcomes

**Figure 3:**
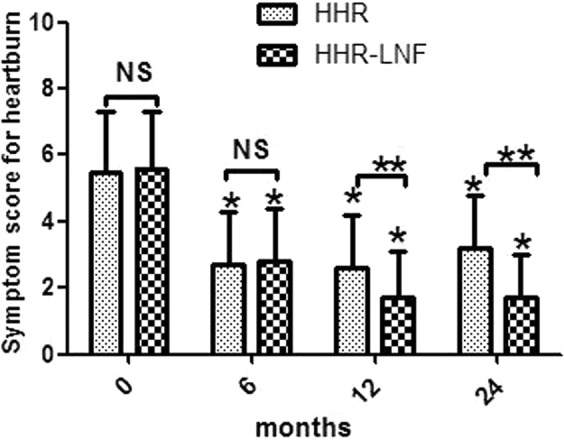
Heartburn score (frequency score + severity score) in selected patients at the indicated time points. The single asterisk (*) and the double asterisk (**) denote statistically significant differences; the single asterisk is for comparison of preoperative and postoperative values, and the double asterisk is for comparison between the two groups (*P* < 0.05). NS = not statistically significant; HHR = laparoscopic hiatal hernia repair; HHR-LNF = laparoscopic hiatal hernia repair with concomitant Nissen fundoplication.

**Figure 4:**
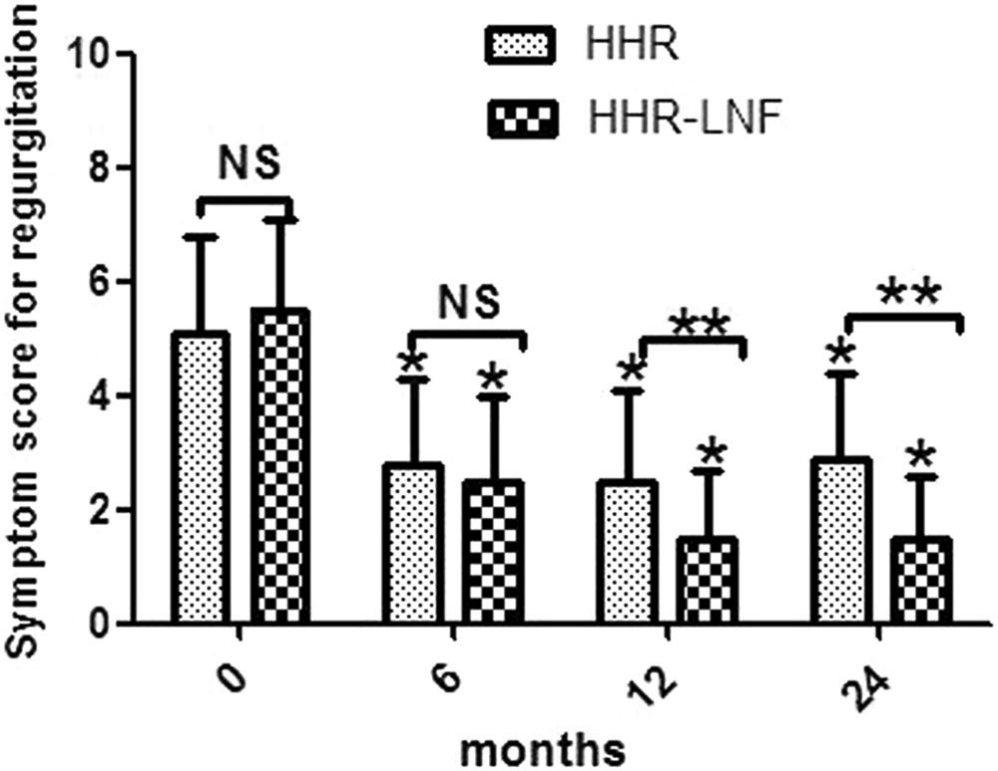
Regurgitation score (frequency score + severity score) in selected patients at the indicated time points. The single asterisk (*) and the double asterisk (**) denote statistically significant differences; the single asterisk is for comparison of preoperative and postoperative values, and the double asterisk is for comparison between the two groups (*P* < 0.05). NS = not statistically significant; HHR = laparoscopic hiatal hernia repair; HHR-LNF = laparoscopic hiatal hernia repair with concomitant Nissen fundoplication.

Preoperatively, Table 1 showed GERD-related symptoms were very common in both groups, with no statistically significant difference between the groups (all P > 0.05). At 6 months after surgery, Figs. 3 and 4 showed that while the HHR and HHR + LNF groups significantly improved over their preoperative values, there was no significant difference between the two groups. However, by 12 and 24 months, the HHR + LNF group always showed significantly less heartburn and regurgitation scores than the HHR group (all *P* < 0.001).

### Patient satisfaction with surgery

81.8% (45/55) of HHR-LNF patients were fully or partially satisfied with the surgical results, but only 47.2% (25/53) in HHR patients (*P* < 0.001). The other 18.2% (10/55) HHR-LNF patients and 52.8% (28/53) HHR patients continued to be symptomatic and needed medication or repeat surgery. Two HHR-LNF patients were rehospitalized for laparoscopic revision (1 for recurrent hiatal hernia and 1 for dysphagia). 20 HHR patients were rehospitalized for LNF. HHR patients had a significantly higher reoperation rate than HHR-LNF patients (*P* < 0.001). No major complications or deaths occurred during the study.

## Discussion

This study was to evaluated the treatment effect of fundoplication as routine adjunct therapy for all types of HHR. Patients with symptomatic HH were assigned by intraoperative central randomization to receive either HHR or HHR-LNF. The 122 patients were randomly assigned. At the time of the latest follow-up, 53 LNF patients and 55 HHR-LNF patients were taken into account in the final analysis (Fig. 1). We assessed the frequency of esophagitis, relative symptom improvement, patient satisfaction, reoperation rate and incidence of complications after laparoscopic antireflux surgery. The patients who would not tolerate or have no choice of a fundoplication were shown to impossibly benefit from a lesser procedure–just a reduction of the hernia and diaphragmatic crura approximation. Our data results could confirm some important points. The presence of HH can cause incompetence of the lower esophageal sphincter^21^, thus increase the occurrence of acidic reflux during a transient relaxation of the lower esophageal sphincter in patients with GERD. Moreover, defective esophageal hiatus could weaken the anti-reflux barrier^22^. Up to 51% of patients with GERD find the presence of HH^23^. The large hernial sac may also exert pressure on the surrounding tissues and lead to recurrent pneumonia, atelectasis, and compression of the atrium and pulmonary vein^24,25^. To avoid the complications and risks of HH, drug refractory patients with HH should undergo early surgery^26^. However, whether fundoplication should be a routine adjunct to HHR in patients with HH is still uncertain. We hypothesized that fundoplication should also be a routine adjunct to HH patients.

In our study, all types of HH were treated by HHR or HHR-LNF. GERD and HH are mainly defined by the symptoms, and so we think that the subjective symptoms of patients are the most appropriate indicators to assess efficacy. Objective evidence of reflux control was obtained by esophageal manometry and 24-hour pH monitoring at 6 months, with added gastroscopic examination at 12 months. The severity of postoperative symptoms and the patient’s quality of life were evaluated using questionnaires. Both procedures—HHR and HHR-LNF—resulted in improvements over the short-term and mid-term (12 months and 24 months), as reflected by the RDQ scores and the decrease in frequency and severity of GERD symptoms such as heartburn and regurgitation.

At follow-up 12 months after surgery, gastroscopy revealed esophagitis in 44.64% of LNF patients vs. 13.79% of HHR-LNF patients. Both the frequency and the severity of esophagitis were significantily greater in HHR group, which were the samilar as a previous study^15^.

We also assessed patient satisfaction with the treatment results. 12 months after the procedure, 81.8% (45/55) HHR-LNF patients and 47.2% (25/53) LNF patients were fully or partially satisfied with the symptom relief following surgery. Luketich *et al*.^27^ found significant improvement in preoperative symptoms after laparoscopic repair of giant paraesophageal hernia; in their study, 90% of patients showed high degree of satisfaction on a GERD health-related quality of life scale, which was the same as in our study.

Postoperative complications mainly occurred in the mean duration of postoperative hospital stay in HHR group, such as dysphagia, abdominal bloating and abdominal pain. These symptoms were due to the occurrence of postoperative esophageal hiatus narrow, poor gastric motility. The esophageal elongation associated with surgery also is a factor. Leaving 2 cm or more of the abdominal esophagus to preserve the lower esophageal sphincter is important factors of the anti-gastroesophageal reflux^28^. The risk of treatment-associated complications did not appear to be increased by the addition of fundoplication to HHR. Although dysphagia, abdominal bloating and abdominal pain occurred with higher frequency in the HHR-LNF group, both perioperatively and at 1 months postoperatively. After symptomatic treatment, there was no difference between the two groups in the incidence of the complications at 6 months. In this study population, only 11.4% (13/114) of patients had postoperative complications in the two groups, this was consistent with the 8%-28% complication rates that had been reported by others^29,30^.

Recurrent HH has been reported to occur following antireflux surgery in 50%-70% of patients with large paraesophageal hernias^31,32^. In our study, a recurrent HH was revealed in only 1.7% (1/58) HHR-LNF group and in 5.4% (3/56) HHR group (P > 0.05). The overall recurrence rates of 3.5% (4/114) was lower than reported in the above literature. Therefore, an concomitant fundoplication maybe could not increase the risk of hernia recurrence when a HHR is performed. Moreover, up to 37.7% (20/53) HHR patients were rehospitalized for LNF, only 3.6% (2/55) HHR-LNF patients were rehospitalized for laparoscopic revision (1 for recurrent hiatal hernia and 1 for dysphagia). HHR patients had a significantly higher reoperation rate than HHR-LNF patient.

With regard to the mechanism of GERD, the lower esophageal sphincter, or valvular mechanism, no matter which is destroyed and it cannot prevent the occurrence of reflux. HH may predict higher risk for GERD due to its negative impact on the esophagus^33,34^. Although both HHR and HHR-LNF patients had significantly improved LESP during the normal value (13–43 mmHg) at 6 months after surgery, patients with HHR-LNF reported significantly fewer reflux symptoms than did HHR patients. Conversely, the procedure of only HHR did not produce improvement in mid-term or long-term reflux symptoms. Therefore, HH just is an important factor in aggravating gastroesophageal reflux; Effective control of reflux mechanisms is the key factors for the successful management of GERD-related symptoms^35^. Fundoplication should be routinely performed in HH patients to reduce the risk of postoperative persistence of GERD or of new-onset GERD.

This study has several limitations. For example, designed methods, excellent randomization and relevant parameters of measurement may be less valuable.

In summary, we found HH patients undergoing HHR-LNF had better midterm outcomes than HHR alone. These data might indicate that early assessment of HH patients, and opting for routine adjunct of fundoplication in HHR, could significantly improve clinical outcomes.
